# Synchrotron-Based Structural Analysis of Nanosized Gd_2_(Ti_1−x_Zr_x_)_2_O_7_ for Radioactive Waste Management

**DOI:** 10.3390/nano15141134

**Published:** 2025-07-21

**Authors:** Marco Pinna, Andrea Trapletti, Claudio Minelli, Armando di Biase, Federico Bianconi, Michele Clemente, Alessandro Minguzzi, Carlo Castellano, Marco Scavini

**Affiliations:** 1 Dipartimento di Chimica, Università degli Studi di Milano, Via Golgi 19, 20133 Milan, Italy; andrea.trapletti@unimi.it (A.T.); armando.dibiase.adb@gmail.com (A.d.B.); federico.bianconi@studenti.unimi.it (F.B.); michele.clemente1@studenti.unimi.it (M.C.); alessandro.minguzzi@unimi.it (A.M.); 2 Dipartimento di Energia, Politecnico di Milano, Via Lambruschini 4a, 20156 Milan, Italy

**Keywords:** synchrotron radiation, complex metal oxides, HR-XRPD, EXAFS, PDF, radiation waste management, Gd_2_(Ti_1−x_Zr_x_)_2_O_7_

## Abstract

Complex oxides with the general formula Gd_2_(Ti_1−x_Zr_x_)_2_O_7_ are promising candidates for radioactive waste immobilization due to their capacity to withstand radiation by dissipating part of the free energy driving defect creation and phase transitions. In this study, samples with varying zirconium content (xZr = 0.00, 0.15, 0.25, 0.375, 0.56, 0.75, 0.85, 1.00) were synthesized via the sol–gel method and thermally treated at 500 °C to obtain nanosized powders mimicking the defective structure of irradiated materials. Synchrotron-based techniques were employed to investigate their structural properties: High-Resolution X-ray Powder Diffraction (HR-XRPD) was used to assess long-range structure, while Pair Distribution Function (PDF) analysis and Extended X-ray Absorption Fine Structure (EXAFS) spectroscopy provided insights into the local structure. HR-XRPD data revealed that samples with low Zr content (xZr ≤ 0.25) are amorphous. Increasing Zr concentration led to the emergence of a crystalline phase identified as defective fluorite (xZr = 0.375, 0.56). Samples with the highest Zr content (xZr ≥ 0.75) were fully crystalline and exhibited only the fluorite phase. The experimental *G*(*r*) functions of the fully crystalline samples in the low *r* range are suitably fitted by the Weberite structure, mapping the relaxations induced by structural disorder in defective fluorite. These structural insights informed the subsequent EXAFS analysis at the Zr-K and Gd-L_3_ edges, confirming the splitting of the cation–cation distances associated with different metal species. Moreover, EXAFS provided a local structural description of the amorphous phases, identifying a consistent Gd-O distance across all compositions.

## 1. Introduction

Complex metal oxides with the general formula A_2_B_2_O_7_ have garnered significant attention due to their wide range of applications, including catalysis, ionic conduction, and nuclear waste immobilization [1,2,3]. In particular, solid solutions with the formula Gd_2_(Ti_1−x_Zr_x_)_2_O_7_, thanks to their remarkable resistance to α-particle radiation-induced amorphization, have attracted considerable interest in radioactive waste disposal [4,5,6]. Studies have shown that increasing the Zr content enhances the structural stability of these materials under irradiation [7,8,9,10]. The mechanism through which these compounds dissipate radiation damage strongly depends on composition. While, in Gd_2_Ti_2_O_7_, radiation damage induces amorphization [7], in Gd_2_Zr_2_O_7_, it is believed to involve a structural transition from the ordered pyrochlore phase to a disordered and defective fluorite structure [11]. This phase transition can be regarded as an order–disorder transition, occurring through the accumulation of structural defects.

In complex structures, the doping process itself can lead to these kinds of phase transitions [11,12,13,14]. To predict the structural stability of synthesized pyrochlores, the ratio between the A and B site cation radii (*r*_A_/*r*_B_) has been widely employed [15,16]. Specifically, for *r*_A_/*r*_B_ < 1.46, the system assumes the structure of an anion-deficient fluorite (i.e., A_0.5_B_0.5_O_1.75_), while in the 1.46 < *r*_A_/*r*_B_ < 1.78 range, the pyrochlore is the most stable phase. Finally, for values of *r*_A_/*r*_B_ > 1.78, a layered monoclinic perovskite structure is found [8,17,18,19].

In this context, the Gd_2_(Ti_1−x_Zr_x_)_2_O_7_ solid solution series, spanning from Gd_2_Ti_2_O_7_ to Gd_2_Zr_2_O_7_, represents an ideal model system to investigate the effect of the A/B ionic radius ratio on structural behavior, as the two end-members exhibit markedly different values, 1.74 for Gd_2_Ti_2_O_7_ and 1.46 for Gd_2_Zr_2_O_7_. The latter value coincides with the boundary between the fluorite and the pyrochlore regimes.

In a previous study, we investigated well-grown samples produced by solid-state synthesis at 1500 °C using High-Resolution X-ray Powder Diffraction (HR-XRPD) and Pair Distribution Function (PDF) analyses, demonstrating that the Ti/Zr concentration strongly affects defect thermodynamics and structural features across different length scales [11]. According to both reciprocal and real-space analysis, Gd_2_Ti_2_O_7_ displays an almost perfect pyrochlore structure. The Rietveld refinements revealed that substituting Ti with Zr leads to the formation of Anti-Frenkel (AF) oxygen defects, whose concentration increases almost linearly with the Zr content (xZr). Additionally, Zr/Gd antisite defects emerge for xZr ≥ 0.75 [11,20]. The *G*(*r*) functions of the Zr-rich samples deviate from the pyrochlore model for *r* < 9 Å and, below 8 Å, they can be satisfactorily fitted either by a Weberite-like structure, in agreement with the literature reports on Ho_2_(Ti_1−x_Zr_x_)_2_O_7_ (space group *Ccmm*) [21], or by ab initio Density Functional Theory (DFT) calculations incorporating clusters of Anti-Frenkel (AF) defects. In addition, DFT calculations revealed that AF defects lower the free energy of Gd_2_Zr_2_O_7_. Specifically, Gd_2_Zr_2_O_7_ (and Zr-rich solid solutions) intrinsically contain a high concentration of defects, and irradiation could further drive the system toward the pyrochlore/defect fluorite boundary. Conversely, the formation of defects in Gd_2_Ti_2_O_7_ is energetically less favorable [22], promoting amorphization rather than facilitating the pyrochlore to defect fluorite transition.

When nanocrystalline Gd_2_Zr_2_O_7_ is synthesized by co-precipitation of mixed hydroxides and treated at temperatures between 600 °C and 1600 °C, it exhibits a behavior depending on the synthesis temperature [23]. According to standard XRPD analysis, the initially amorphous precursor first transforms into a fluorite-like phase and eventually crystallizes into the pyrochlore structure. In contrast, Gd_2_Ti_2_O_7_, prepared by the sol–gel route, crystallizes directly into the pyrochlore phase at 800 °C, with no intermediate fluorite phases detected by XRPD [24].

All these findings suggest that the degree of structural ordering in these materials is influenced not only by chemical substitutions, but also by synthesis conditions and thermal treatments. Furthermore, a strong correlation exists between the disordering mechanisms occurring at decreasing calcination temperatures and progressive irradiation-induced amorphization of initially crystalline compounds, as evidenced in the literature, for example by X-ray Near Edge Absorption Spectroscopy (XANES) results at the Ti K-edge [24,25].

In other words, amorphous or nanocrystalline materials can mimic the crystal structure of irradiated gadolinium zirconates and titanates, providing valuable structural information on their defect arrangements without the need to handle samples containing embedded radionuclides.

Therefore, this study aims to perform a structural characterization of a set of samples representative of real matrices exhibiting radiation-induced damage as a result of prolonged alpha decay. Particular attention is devoted to elucidating the influence of chemical composition on the resulting structural modifications, and hence on the material’s simulated response to the high-energy deposition associated with alpha-emitting decay processes.

For this purpose, we chose to prepare nanosized samples with xZr = 0.00, 0.15, 0.25, 0.375, 0.56, 0.75, 0.85, and 1.00 by employing a modified Pechini sol–gel method. This ensured homogeneity of the obtained solid solutions, while avoiding possible contaminations during multiple grinding and annealing steps necessary in the traditionally employed solid-state synthesis [11]. Moreover, the use of the sol–gel polymerization route allowed us to maintain control over the size of the obtained powders. Specifically, this procedure allowed us to obtain nanopowders (with an average diameter < 5 nm). Interestingly, it seems that nanosized complex metal oxides exhibit a different behavior on the local and average scale. Specifically, the use of Extended X-ray Absorption Fine Structure (EXAFS) and PDF to probe the local structure of nanosized Gd_2_Zr_2_O_7_ demonstrated the presence of local distorted interatomic distances [24,26], in contrast with the average defect fluorite structure observed by the Rietveld refinement [23].

Since the understanding of the local and average structure of complex metal oxides is essential to study the energy dispersion phenomena, we opted for a multiscale structural characterization by X-ray Absorption Spectroscopy (XAS) and High-Resolution X-ray Powder Diffraction coupled to Pair Distribution Function analysis. Specifically, EXAFS analysis was performed to study the local structure of our materials, while the Rietveld refinements allowed us to study the long-range structure of the Gd_2_(Ti_1−x_Zr_x_)_2_O_7_ series. Finally, PDF bridged the local and long-range results, revealing the structural reorganization induced by defects in the nanometer range. To the best of our knowledge, this is the first multiscale study on the complete series synthesized via the sol–gel route using synchrotron radiation techniques.

## 2. Materials and Methods

### 2.1. Complex Metal Oxide Synthesis

The Gd_2_(Ti_1−x_Zr_x_)_2_O_7_ samples were synthesized via a modified Pechini sol–gel method. Gadolinium nitrate hexahydrate (Gd(NO_3_)_3_∙6H_2_O, 99.9%, Sigma-Aldrich, St. Louis, MO, United States of America), titanium isopropoxide (Ti[OCH(CH_3_)_2_]_4_, 97%, Sigma-Aldrich, St. Louis, MO, United States of America), and zirconium butoxide (Zr(OC_4_H_9_)_4_, 80% wt. in n-butanol, Sigma-Aldrich, St. Louis, MO, United States of America) were used as metal precursors.

The titanium and zirconium alkoxides were first dissolved in Milli-Q water (18.2 MΩ·cm^−1^ at 25 °C, obtained using a Millipore (MilliporeSigma, Burlington, MA, USA) purification system), along with citric acid (C_6_H_8_O_7_, ≥99.5%, Sigma-Aldrich, St. Louis, MO, USA) as a chelating agent, keeping a 1:20 molar ratio of tetravalent metal cations to citric acid. Ethylene glycol (C_2_H_6_O_2_, ≥99.5%, Honeywell Fluka, Seelze, Germany) was then added in a 2:1 molar ratio with respect to citric acid, followed by the stoichiometric amount of Gd nitrate to complete the metal precursor mixture.

The resulting solution was stirred continuously and heated on a hot plate until the release of nitrate vapors was observed and a viscous brown gel formed. Upon complete solvent evaporation, the resulting polymer was transferred to a muffle furnace (Nabertherm GmbH, Lilienthal, Germany) and calcined in air at 773 K for 3 h using a ramp rate of 4 K·min^−1^ to remove organic residues and obtain nanosized powders.

### 2.2. In-House Characterization

Morphological characterization was performed using Scanning Electron Microscopy (SEM) analysis employing a TM1000 SEM (Hitachi Inc., Tokyo, Japan), and Energy Dispersive X-ray Spectroscopy (EDX) analysis was performed using a TM X-stream instrument (Oxford Instrument, Oxford, UK).

### 2.3. HR-XRPD and PDF Analysis

HR-XRPD patterns were collected during experiment CH6375 at the ID22 beamline of the ESRF. Analysis was performed at 90 K (using a cryostream), to reduce the thermal vibration, on powder samples loaded in Kapton^®^ tubes (diameter = 1 mm) and mounted parallel to the diffractometer axis using a transmission geometry.

XRPD patterns were collected employing an X-ray beam with a horizontal and vertical size of 1.0 mm × 0.9 mm and a wavelength of *λ* = 0.354408(2), as determined using a Si standard; the high-resolution setup of the ID22 beamline was employed, which features a 13-channel Si 111 multi-analyzer stage between the sample and the Dectris Eiger 2 X2M-W detector (Dectris Ltd., Baden-Dätwill, Switzerland) [27]. The 2Θ intervals ranged from 3 < 2Θ < 50° for the Rietveld refinements to 3 < 2Θ < 120° for PDF analysis. In the latter case, multiple scans were averaged for a total counting time of ≈2 h. The lack of radiation damage was confirmed comparing the first and the last scans of each pattern. PDF quality data were collected on selected samples, i.e., GTZ_0_, GTZ_37.5_, GTZ_56_, GTZ_75_, and GTZ_100_ and an empty Kapton^®^ tube, for background subtraction.

Concerning the reciprocal-space Rietveld analysis, the structural models were fitted in the reciprocal space using GSAS suite of programs for the Rietveld analysis and its graphical interface EXPGUI [28,29].

In addition, we employed the Williamson–Hall method to calculate the average crystallite size [30].

For real-space analysis, we used the reduced PDF, *G*(*r*), obtained from the sine Fourier transform of the experimental total scattering function, *S*(*Q*), as defined in Equation (1) [31]:

(1) $$G\left(r\right)=4\pi r\left[\rho \left(r\right)-\rho_{0}\right]=\frac{2}{\pi }\int_{Q_{min}}^{Q_{max}}Q\left[S\left(Q\right)-1\right]\sin\left(Qr\right)dQ$$

where $\rho \left(r\right)$ is the atomic pair density function and indicates the probability of finding an atom at a distance *r* from another atom, while $\rho_{0}$ is the atom number density. *G*(*r*) curves were computed using the PDFgetX3 program [32] using data up to *Q_max_* = 27 Å^−1^. Structural models were fitted to the *G*(*r*) curves using PDFgui [33].

### 2.4. EXAFS Analysis

EXAFS measurements were performed in transmission geometry at the BM08 beamline of the ESRF during experiment CH6416 [34]. Samples were analyzed at the Gd L_3_-edge (7242 eV) and Zr (17,996 eV) K-edge, using ionization chambers as detectors. A Si crystal cut along the (311) plane was used as monochromator. Calibration was performed using a metallic Zr foil as the reference standard for the Zr K-edge, and a metallic Fe foil for the Gd L_3_-edge. The latter choice was made due to the proximity of the Fe K-edge (7112 eV) and the unavailability of a metallic Gd standard. Pt-coated mirrors were used to reject the harmonics at the Zr K-edge, while Si-coated ones were used at the Gd L_3_-edge [34].

For transmission measurements, samples were mixed with cellulose by grinding in an agate mortar and uniaxially pressed into 1.3 cm diameter pellets with a hydraulic press. The amount of sample required to get an optimal jump at the absorption edge was calculated employing XASmass [35]. Data were acquired in isothermal conditions at room temperature (300 K) and at 100 K. Cooling was provided by using an “Oxford instrument” nitrogen flow cryostat.

EXAFS data were reduced by employing the Demeter package [36], while fits of the k^2^-weighted data were carried out in *r* space using theoretical functions from the FEFF9 code [37].

To calculate phases and amplitudes of the Feynman path, we employed two different CIFs, provided by the Materials Project [38]: a cubic zirconia ZrO_2_ [39] for the Zr K-edge and a cubic GdO [40] for the Gd L_3_-edge. Specifically, to overcome the inability of atoms to treat doped samples, the atomic positions computed from those CIFs were manually modified in order to describe a cubic structure where the cationic positions were half-occupied by the absorber and half by a different cation (Gd and Ti for the Zr K edge; Zr and Ti for the Gd L_3_ one). For every sample, we included the first shell A-O(1) (A: absorber) and all the A-M(1) (M: metal) distances. So, those samples containing both Ti and Zr needed an additional path with respect to the endmembers. The stoichiometry of the undoped samples (GTZ_0_ and GTZ_100_) can be easily achieved by setting the correct value of the occupation number: it is 7 in X-O(1) paths (in order to reproduce the defective fluorite) and 6 in X-M. Doping can be introduced by controlling the inelastic factor: due to a software limitation, the occupation number cannot be fractional, but since it is multiplied by S_0_^2^, we can reproduce the same effect of partial occupation by reducing this value. So, the stoichiometry of GTZ_x_ samples (x = 85, 75, 56) is maintained simply by setting the value of S_0_^2^ equal to the concentration of the relative species.

For each path, the initial fitting parameters were the mean-square disorder in the neighbor distances σ^2^ and the interatomic distances r. We also considered a single, common value of energy shift E_0_ with respect to the theoretical value.

## 3. Results and Discussion

### 3.1. In-House Characterization of Complex Metal Oxides

For the sake of brevity, samples will be hereafter referred to as GTZ_x_, where x represents the amount of dopant (i.e., xZr) in the material.

The sample morphologies were studied using Scanning Electron Microscopy (SEM). As shown in Figure S1, no significant differences are present for differently doped samples. All samples appear as conglomerates of nanoparticles too small to be detected by the employed technique. As for the composition, we notice that the ratio between Zr and Ti in the studied Zr-doped samples (Table 1) is close to the nominal one based on the complex metal oxide stoichiometry. For completeness, we report all analyzed spectra, along with those of pure Gd_2_Ti_2_O_7_ (GTZ_0_) and Gd_2_Zr_2_O_7_ (GTZ_100_), in the Supporting Information (Figure S2).

### 3.2. Average Structure

The average structure of the GTZ samples was investigated using HR-XRPD analysis and the Rietveld refinements. Since the high-resolution diffraction patterns were to be employed to obtain information on the local structure by PDF analysis, measurements were performed at 90K. This temperature was chosen to limit the atoms’ thermal motion and thus focus on structural disorder.

**Figure 1:**
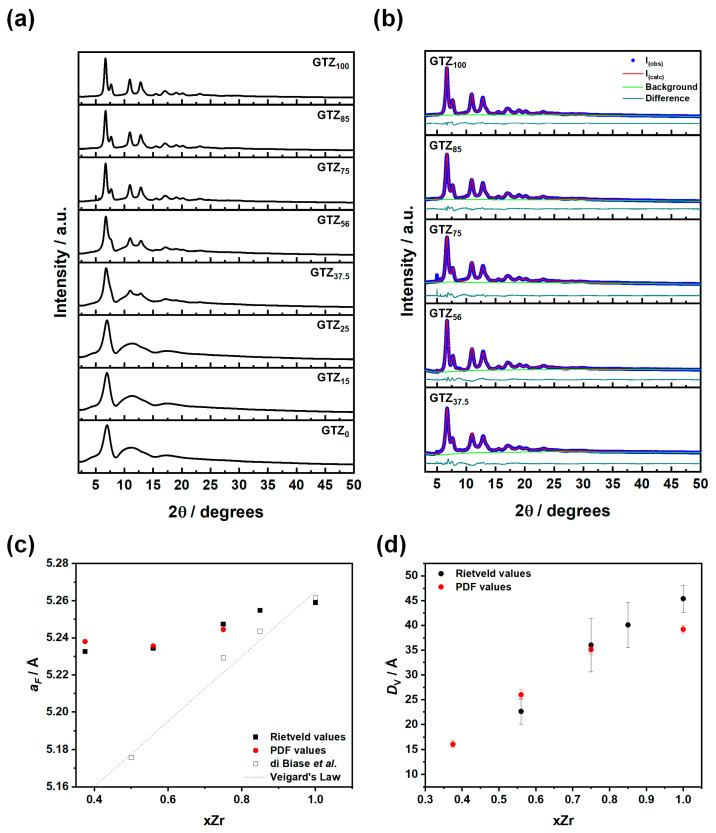
In (**a**), we show the diffraction patterns of the samples studied, while in (**b**), we report the Rietveld refinements of the same patterns. In (**c**), the lattice parameter *a_F_*, according to the fluorite model, is shown as a function of Zr content in the GTZ_x_ samples (□ data source: [11]). Finally, in (**d**), we present the Williamson–Hall results.

As shown in Figure 1a, pure Gd_2_Ti_2_O_7_ and the Gd_2_(Ti_1−x_Zr_x_)_2_O_7_ solid solutions (xZr = 0.15 and 0.25) with the highest Ti content are fully amorphous. Apart from the first sharp diffraction peak [41] at 2θ ≈ 6.95°, only broad bands appear in the patterns. With increasing Zr content (xZr = 0.375 and 0.56), the Bragg peaks begin to emerge alongside the amorphous signal. Finally, for the solid solutions with the highest Zr content (xZr = 0.75 and 0.875) and pure Gd_2_Zr_2_O_7_, the amorphous phase disappears entirely, leaving only the Bragg peaks. These peaks are present at 6.72, 7.76, 11.00, 12.90, 15.57, 16.97, 19.10, 20.27, and 23.11 degrees and correspond to the 111, 200, 220, 311, 400, 331, 422, 333, and 531 reflections of the (defect) fluorite $Fm\bar{3}m$ structure (SG: 225).

Within this model, Gd, Ti, and Zr fully occupy the 4a (0, 0, 0), while O is positioned in the 8a (¼, ¼, ¼) Wyckoff positions (occupation factor = 7/8). We performed the Rietveld refinements to obtain structural information on the GTZ samples; the refinements and the refined results are reported in Figure 1b and Table 2, respectively. To reduce correlation with the background, we blocked the refinement for the mean square displacement values (U_ave_) to be the same for all atoms. In the case of GTZ_56_ and GTZ_37.5_, we subtracted the pattern of amorphous GTZ_25_ to extract the peaks. Figure S3 shows the superimposed original diffraction patterns of the samples used for the subtraction procedure. Figure 1c reports the calculated cell constants (black squares) as a function of the Zr-doping content xZr. In the same figure, the same parameters as calculated using PDF analysis (red circles, see below) and the results obtained on microcrystalline Gd_2_(Ti_1−x_Zr_x_)_2_O_7_ samples (empty squares), taken from our own previous work, are also reported for the sake of comparison [11].

Upon reducing the Zr concentration, the cell parameter *a_F_* shrinks, as shown by both reciprocal-space (Rietveld) and real-space (PDF) analysis. This is consistent with the substitution of the larger Zr^4+^ ion (0.72 Å) by the smaller Ti^4+^ ion (ionic radius of 0.605 Å) [42]. However, unlike the microcrystalline case, the cell constants of the nanocrystalline samples do not follow a linear trend. In particular, transitioning from fully crystalline samples to partially amorphous ones, the cell parameter tends to a constant value. This suggests that the biphasic samples are inhomogeneous not only crystallographically but also compositionally: the amorphous phase is richer in Ti, while the nanocrystalline phase is poorer than the average composition. Additionally, we note that, apart from the GTZ_100_ case, the cell constants of the nanocrystalline samples are larger than those of the microcrystalline ones. This is a well-known effect observed in fluorite nanoparticles and is attributed to surface relaxation [43,44,45].

To calculate the crystallite size *D*_V_ and the inhomogeneous strain parameter ε, we employed the Williamson–Hall method [46]. Reliable *D*_V_ and ε parameters have been calculated only for the fully crystalline samples because, in the patterns of the biphasic samples (fluorite + amorphous, xZr = 0.375 and 0.56), the first sharp diffraction peak of the amorphous phase and the 111, 200 peaks of the fluorite phase overlap, increasing their broadening. In particular, for the GTZ_37.5_ sample, the ε parameters took an unphysical negative value. For this reason, we omitted the fitted parameters for this composition in Table 2. As shown, ε is very high (≈10^−2^) for all fully crystalline samples, highlighting the strongly strained nanocrystalline nature of the particles obtained. As shown (black symbols in Figure 1d), with the decrease in Zr content, the average crystal size decreases. This trend is also confirmed by the analysis of the *G*(*r*) functions described below (red circles, Figure 1d).

### 3.3. Local Structure

#### 3.3.1. PDF Results

**Figure 2:**
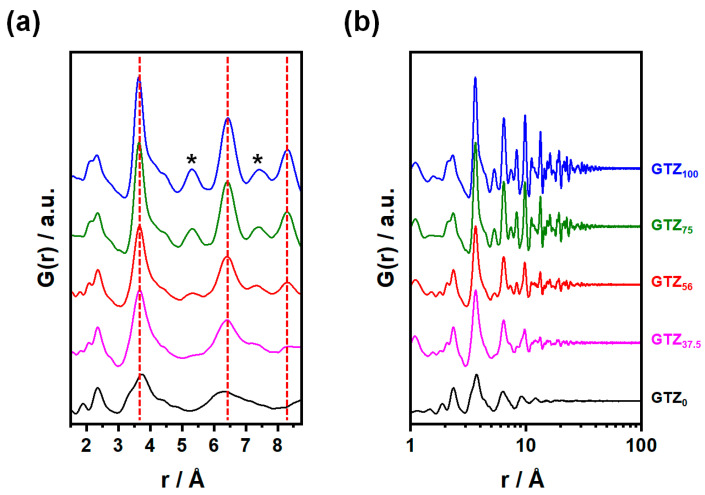
In (**a**), we present the short-range region, while in (**b**), we show the full *G*(*r*) functions for GTZ_100_ (blue line), GTZ_75_ (green line), GTZ_56_ (red line), GTZ_37.5_ (pink), and GTZ_0_ (black line). The symbol * represents additional metal-metal distances of interest, details are provided in the text.

To highlight the local structure, we report in panel a of Figure 2 the short-range region (i.e., *r* < 8 Å) of the experimental PDFs collected for selected samples. The peaks below ≈2.5 Å are attributed to the cation–oxygen interatomic distances. Starting from pure Gd_2_Zr_2_O_7_, the peaks at *r* ≈ 2.1 Å and 2.35 Å can be attributed to Zr-O and Gd-O bond distances, respectively. This is consistent with the values calculated by Popov and coworkers using EXAFS measurements at the Zr-K and Gd-L_3_ edges on a similarly annealed sample [23], as well as with the results presented in Section 3.3.2 of the present work. Increasing the Ti content in the solid solution system leads to a decrease in the intensity of the peak at 2.1 Å, and its complete disappearance in pure Gd_2_Ti_2_O_7_. In contrast, the peak at 2.35 Å remains almost unaffected by composition. In the samples with the highest Ti content, a new peak appears and increases with Ti content at *r* ≈ 1.9 Å, which is attributed to Ti-O distances, in agreement with the literature [24].

The vertical red dashed lines correspond to the most intense cation–cation distances in this *r* range, according to the fluorite structure. Two other cation–cation distances are labeled with asterisks. Starting from the Gd_2_Zr_2_O_7_ sample, the peak at ≈3.62 Å is sharp with a tail on its right side. This tail, already observed in the PDF of microcrystalline Gd_2_Zr_2_O_7_ samples exhibiting a pyrochlore structure [11,23], is discordant with the fluorite structure and suggests the presence of local disorder. When lowering the Zr concentration, the peak broadens but does not significantly shift in position, down to xZr = 0.375. In Gd_2_Ti_2_O_7_, the peak maximum shifts toward larger distances (=3.73 (7) Å), and an additional tail appears on its right side (=3.30 (9) Å). Sigma values are obtained by fitting with two Gaussian functions.

Moving to larger interatomic distances, similar trends are observed as the sample composition changes: the peaks progressively broaden while maintaining approximately the same position, at least down to a Zr concentration of xZr = 0.375. Additionally, in the *G*(*r*) function of the latter, the peaks at ≈5.3 Å and 7.4 Å almost disappear. We recall that the samples with xZr = 0.56 and 0.375 are only partially (nano)crystalline, while Gd_2_Ti_2_O_7_ appears to be completely amorphous. Accordingly, for this last sample, only broad modulations of the *G*(*r*) function appear above ≈5 Å. Since the broadening of *G*(*r*) peaks is related to the distribution of interatomic disorder, we note that disorder increases with the addition of Ti to the structure, which is the opposite of what was observed in well-grown microcrystalline samples [11].

Figure 2b displays the same PDFs over a wider *r* range, using a logarithmic scale for the abscissa. The amplitude of all *G*(*r*) function decays within 4–5 nm, with a steeper rate for the samples with the highest Ti content, confirming the nanostructured (and even amorphous) nature of the samples.

**Figure 3:**
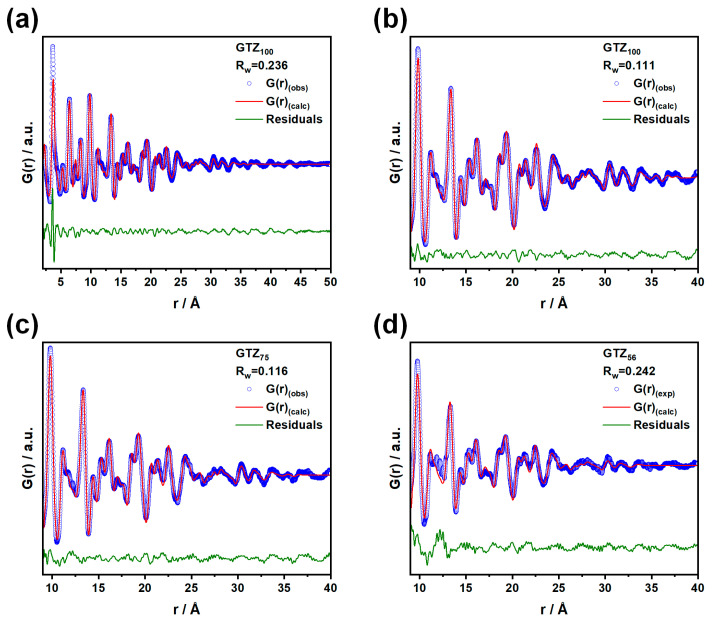
In (**a**), the full-range PDF fit of GTZ_100_ is shown using the defect fluorite model, while in (**b**), the fit is limited to the 9–40 Å range. The same range and fitting method are applied to (**c**) GTZ_75_ and (**d**) GTZ_56_, respectively. In all panels, the empty blue circles represent the experimental data, the red line shows the calculated *G*(*r*), and the green line represents the residuals.

To calculate the crystallographic coherence lengths of the nanocrystals, the experimental *G*(*r*) of Zr-containing samples was fitted using the real-space Rietveld analysis, and the fluorite structure was adopted over a wide *r* range (1.9 ≤ *r* ≤ 50 Å), varying the cell parameter, the atomic mean square displacements U_M_ for Gd, Zr, Ti and U_O_ for oxygen, a scale factor, and the particle diameter *D*_V_. Table S1 in the Supporting Information reports the refined parameters, while Figure 3a shows the fit for Gd_2_Zr_2_O_7_ as an example. Discrepancies between the experimental data and the fits are particularly evident in the low-r region for all samples, indicating that the average fluorite structure hides a significant degree of local disorder (Figure S4). However, PDF analysis confirms the same trend in crystallite size detected by W-H analysis: as the Zr concentration decreases, *D*_V_ ranges from ≈39 Å for Gd_2_Zr_2_O_7_ to ≈16 Å for xZr = 0.35 (see red circles in Figure 1d).

Since the largest discrepancies between the experimental data and the fluorite model appear at low *r* values, we analyzed the short-range data (up to 8 Å) separately from the range between 9 Å and 40 Å.

We show the fits for Gd_2_Zr_2_O_7_ in panel b of Figure 3, while the fits for xZr = 0.75 and xZr = 0.56 are reported in panels c and d, respectively.

In the cases of Gd_2_Zr_2_O_7_ (Figure 3b) and Gd_2_(Zr_0.75_Ti_0.25_)_2_O_7_ (Figure 3c), we observe an acceptable match between the experimental data and the fitted fluorite model, considering the small amplitude of the *G*(*r*) peaks of the nanostructured oxides above ≈1 nm and the approximations introduced: spherical monodispersed nanoparticles without surface relaxations. The presence of significant structural disorder is confirmed by the high values of the U parameters: U_M_ ≈ 0.04 Å^2^ for the metals and U_O_ ≈ 0.14 Å^2^ for oxygen. For xZr = 0.56 (Figure 3d), the fit worsens, and a modulation appears in the residuals that resembles the broad features observed in the experimental PDF of the amorphous Gd_2_Ti_2_O_7_ sample. The same modulation also appears in the residuals of the PDF of the xZr = 0.375 sample (Figure S5).

Due to the complex biphasic nature (nanocrystalline plus amorphous) of the samples with xZr = 0.375 and 0.56, we will limit the short-range data analysis (1.9 ≤ *r* ≤ 8 Å) to the fully crystalline xZr = 0.75 and 1.00 samples. In the following section, we will discuss the simplest case (the Gd_2_Zr_2_O_7_ compound), though the results qualitatively apply to the xZr = 0.75 solid solution as well.

**Figure 4:**
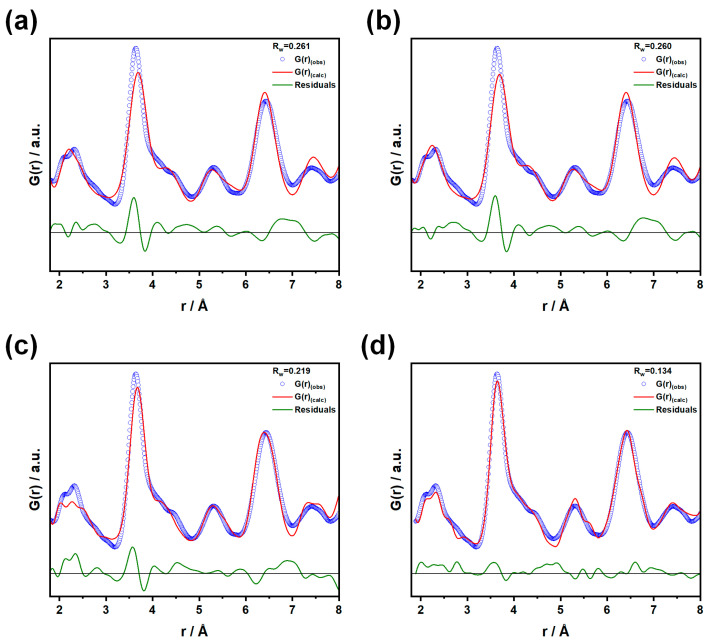
We report the fits for GTZ_100_ using (**a**) a fluorite model, (**b**) the defect fluorite, and (**c**) the defect model including Anti-Frenkel defects, both from our own previous study [12]. Finally, in (**d**), we report the Weberite model.

First, we adopted the fluorite model, fixing the *D*_V_ parameter to the value refined over the larger *r* range. The fit, shown in panel a of Figure 4, reiterates all the misfits observed above. In a second attempt, shown in panel b of Figure 4, we employed the pyrochlore structure, derived from the defect fluorite structure by the ordering of cation and oxygen vacancies, corresponding to the long-range structure of microcrystalline Gd_2_Zr_2_O_7_ [11,47,48]. However, this approach did not lead to any appreciable improvement in fit quality (Rw = 0.260). This mismatch between the experimental data and the pyrochlore model is not unexpected, as the experimental *G*(*r*) functions clearly display evidence of complex cation–cation distance distribution, while in the pyrochlore model all cation–cation distances are identical.

In our previous XRPD/PDF investigation on microcrystalline samples, it was found that a high concentration of Anti-Frenkel (AF) defects led to an increase in the Zr coordination number (≈6.4) with respect to the expected value in the perfect pyrochlore structure (=6), along with strong structural relaxations that induced a distribution of the cation–cation distances [11]. To rationalize the PDFs, two different strategies were adopted. In the first case, AF defects were introduced into the structure by moving oxygen ions from the crystallographic O1 site of the pyrochlore structure, where oxygen is coordinated by two Zr^4+^ and two Gd^3+^ ions, to the “interstitial” O3 site with OZr_4_ coordination. Then, structural relaxation was allowed in DFT ab initio calculations, and the defect configurations displaying the lowest energies were selected for comparison to the experimental PDF, demonstrating that extended clusters of Anti-Frenkel defects form in microcrystalline Gd_2_Zr_2_O_7_. As shown in panel c of Figure 4, attempts to apply the same defect model to the present data fail, suggesting that the defect structure of the nanostructured sample could differ significantly from the microcrystalline ones.

In the second case (Figure 4d), a Weberite structure (space group *Ccmm*) was adopted to fit the experimental data. Similarly to the pyrochlore structure, the Weberite structure is derived from the defect fluorite structure, but with a different cation ordering. Specifically, one cation site with 8-fold coordination (Wyckoff letter 4b) is occupied by Gd^3+^, a second site with 6-fold coordination (Wyckoff letter 4a) is occupied by Zr^4+^ (also by Ti^4+^ in solid solutions), and finally, a third site with 7-fold coordination (Wyckoff letter 8g) is shared equally by Gd^3+^ and Zr^4+^. A more detailed description is provided in the literature by di Biase et al. and Shamblin et al. [11,21]. The Weberite structure has been adopted by several authors to describe the short-range features of *G*(*r*) functions in RE_2_(Zr,Ti)_2_O_7_ samples (RE = rare earths) with either fluorite or pyrochlore average structure [11,21,49]. This model allows for an increased average coordination number of Zr (=6.5 in GTZ_100_) and introduces additional positional degrees of freedom that enable better tuning of the cation–cation distances. In panel d of Figure 4, we report the fit using this model. Although the residuals are still high (Rw = 0.133), which could still be partially attributed to the approximations discussed above, improvements are observed, both for the first cation–oxygen distances and the most intense cation–cation ones.

A better fit is obtained for the GTZ_75_ sample (R_w_ = 0.116), as shown in Figure S6. In this case, Gd occupies all the 4b sites, Gd and Zr share the 8g ones (50% each), while Ti and Zr share the 4a sites (50% each). In this way, 75% of Zr^4+^ ions adopt the ideal 7-fold coordination.

The use of the *Ccmm* Weberite model warrants some discussion. It was originally proposed by Shambling and coworkers as the local structure of Ho_2_Zr_2_O_7_, which adopts an average fluorite structure [21]. Using this model, the authors could fit their neutron PDFs up to 15 Å (that is about twice the length of the *b* and *c* axes, ≈7 Å). Since a group/subgroup relationship exists between the $Fm\bar{3}m$ and Ccmm models, a cooperative symmetry breaking of the average fluorite structure was proposed, consisting of cation and anion ordering, with an average coherence length of ≈1.5 nm. In the present case, attempts to extend the Weberite model above 8 Å, that is less than the length of the Weberite *a* axis (≈10 Å), led to severe worsening of the fit. Thus, unlike Shambling and coworkers, we propose the Weberite structure here as an effective tool to describe some important features of structural disorder in Zr-rich Gd_2_(Ti_1−x_Zr_x_)_2_O_7_ nanoparticles. In particular, it enables tuning of the coordination numbers of the involved cations, which, according to the defect fluorite structure, should all be 7-fold-coordinated, independently of the element or xZr. According to the Weberite model, in GTZ_100_, the average coordination numbers for Zr^4+^ ions and Gd^3+^ are 6.5 and 7.5, respectively. In GTZ_75_, these values are 6.75 and 7.5, respectively, while all Ti^4+^ ions are 6-fold-coordinated. The agreement between data and model suggests that, taking the defect fluorite structure as a reference, the tendency to allocate vacancies in the first coordination shell of cations follows a trend: Ti^4+^ > Zr^4+^ > Gd^3+^. This vacancy ordering is accompanied by a significant change in the cation–cation distances, which span broad ranges: 3.56–3.91 Å for the (4b) site, 3.53–3.84 Å for the (8g) site, and 3.53–3.91 Å for the (4a) site. Additionally, a particularly long Gd(4a)-Gd/Zr(8g) distance (≈4.16 Å) is present (see Table S2). Interestingly, for all the three sites the average cation–cation distance is ≈3.7 Å, corresponding to the same distance in the defect fluorite model ≡22aF.

#### 3.3.2. EXAFS Results

**Figure 5:**
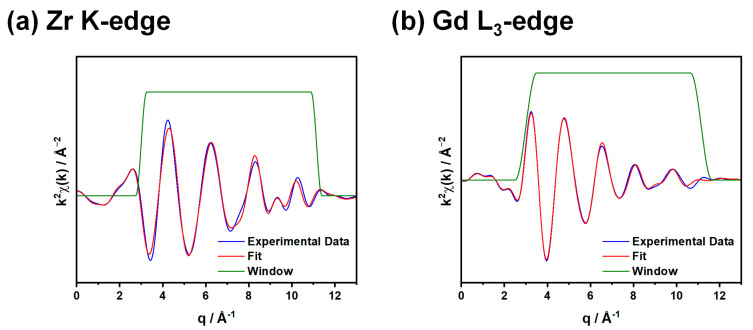
EXAFS fitting at the (**a**) Zr K-edge and (**b**) Gd L3-edge for the GTZ100 sample. In both panels, the experimental data are shown as a blue line, the fitted data are shown as a red line, and the Hanning window used for fitting is displayed in green.

EXAFS data were collected at beamline BM08 of the ESRF at both the Zr K- (17,996 eV, Figure S7a) and Gd L_3_- (7243 eV, Figure S7b) edges during experiment CH-6416. In order to obtain a detectable absorption edge, only GTZ_x_ samples with xZr = 0.75, 0.85, and 1.00 were investigated at the Zr K-edge (Figure 5a), corresponding to the single-phase crystalline samples. This issue did not impact Gd absorption, allowing us to acquire EXAFS data for samples richer in Ti (x = 0.00, 0.56, 1.00) at the Gd L_3_-edge (Figure 5b). In this case, the sample compositions span from fully amorphous (GTZ_0_) to fully crystalline (GTZ_100_).

Due to the proximity of different L-edges among lanthanoid elements, the Gd L_3_ spectra were analyzed within the 3.0–11.15 Å^−1^ k-range (Figure 5) and the 1.2–4.0 Å R-space range (Figure S7c,d). To compare the results obtained from different spectra of the same sample (GTZ_100_), the Zr K spectra were also truncated to the same k and R values. This approach follows Stern’s rule, which emphasizes the importance of optimizing the integration intervals to reduce the risk of correlation within parameters during refinements [50].

To fit the experimental data, either three or four coordination shells were considered for the pure compounds and solid solutions, respectively; these include the following: X-O, X-Gd, X-Zr and/or X-Ti, (X = Zr, Gd). Each coordination number was calculated based on the defect fluorite structure: each cation is surrounded by 7 oxygen atoms, 6 Gd, and 6 Zr/Ti ions. Table S3 reports the refined parameters. If the crystalline samples followed the defect fluorite model at the local scale, all X-O and X-X distances should be ≈2.27–2.28 Å and ≈2.70–2.72 Å, respectively, depending on the sample composition. It is to be noted that this strategy differs from the one adopted for PDF analysis, where a complex model, such as the Weberite structure, involving two different cation sites for both the Gd and Zr elements (and many different M-O, M-Gd/Zr/Ti distances, as listed in Table S3), was applied to the experimental *G*(*r*) functions in the 1.9–8.0 Å range. For this reason, comparisons between the diffraction and absorption results might potentially lead to some partial inconsistencies due to the different approaches used.

##### Zr K-Edge

**Figure 6:**
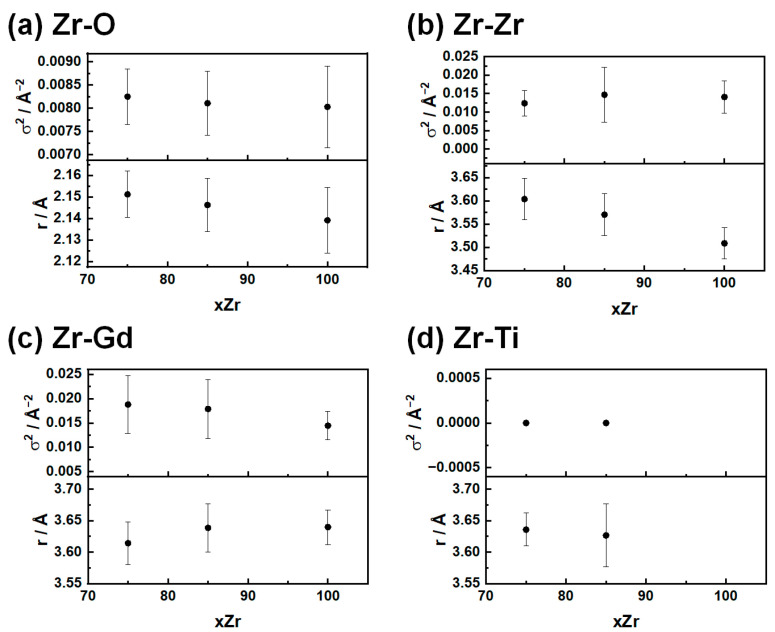
In panels (**a**–**d**), the radial distances are presented, as obtained by the fitting of EXAFS spectra for the (**a**) Zr-O, (**b**) Zr-Zr, (**c**) Zr-Gd, and (**d**) Zr-Ti distances.

Starting with the first coordination shell (Zr-O) surrounding the absorber atom (Figure 6a), we observe that while the distortive term σ^2^ is unaffected by the presence of Ti, there is an apparent contraction of the interatomic distance as a consequence of the reduced Ti occupation of the cationic site. The absolute values are in agreement with the peak at *r* ≈ 2.10 Å observed in the experimental *G*(*r*).

The Zr–Zr distance (Figure 6b) is the shortest interatomic distance involving cation backscatters for all the samples, and it is significantly contracted compared to the value expected from the defect fluorite structure. Increasing Zr concentration leads to a contraction of the Zr-Zr interatomic distance from 3.60 Å in GTZ_75_ to 3.51 Å in GTZ_100_, while the opposite trend (to a smaller extent) is observed for the Zr-Gd bond: from 3.61 Å to 3.64 Å (Figure 6c). The observed differences between Zr-Gd and Zr-Zr distances confirm the PDF findings: the defect fluorite structure cannot account for the local structure of the crystalline GTZ compounds. Additionally, EXAFS reveals that the difference between Zr-Gd and Zr-Zr distances increases with increasing xZr.

Finally, Zr-Ti interatomic distances (Figure 6d) seem to be unaffected by the amount of Ti in the structure for the investigated samples. It is important to note that the value of σ^2^ was set to be null. This additional constraint was applied due to two reasons: without it, the value would be negative, although the error would be large enough to reverse the sign. Since the value must be defined as positive, a negative value cannot be considered. So, we tried setting it as zero, and this test resulted in modifications only to parameter errors, but not to the parameters themselves.

##### Gd L_3_-Edge

**Figure 7:**
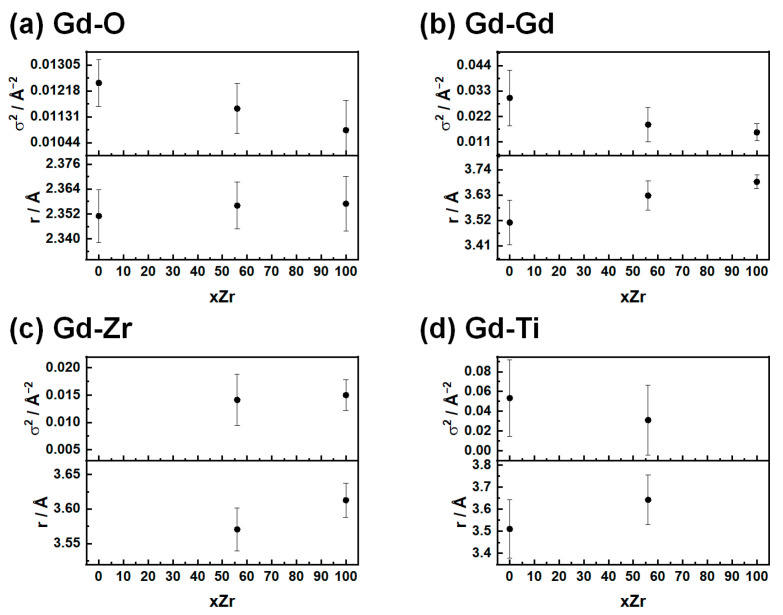
In panels (**a**–**d**), the extracted radial distances are presented, as obtained by the fitting of EXAFS spectra for the (**a**) Gd-O, (**b**) Gd-Zr, (**c**) Gd-Ti, and (**d**) Gd-Gd distances.

The first coordination sphere of the Gd cation is made of oxygen (Figure 7a). The corresponding interatomic distance shows no apparent dependence on the tetravalent cation, at least within the experimental error, and assumes an average value in line with the PDF results (*r* ≈ 2.35 Å). On the other hand, the distortion parameter σ^2^ decreases significantly from the fully amorphous (GTZ_0_) to the fully crystalline phase (GTZ_100_). The same trend is also observed for the Gd-Gd distances, whose weights in the refinement remain the same across the whole compositional range (six Gd-Gd distances on average for each Gd absorber). Since all spectra were collected at the same T (=100 K), the observed changes in σ^2^ values reflect the increasing disorder of these shells when transitioning from the fully crystalline to the fully amorphous phase and are consistent with the broadening of the *G*(*r*) peaks displayed in Figure 2.

As already seen in the case of Zr, the cationic coordination sphere centered on the position of Gd atoms presents different distances. The most interesting behavior is observed in the Gd-Gd distance (Figure 7b): the interatomic distance strongly increases when the system changes from an amorphous phase to a fluorite-like phase (from 3.51 Å to 3.69 Å).

The values of parameters describing the Gd-Zr and Gd-Ti shells (Figure 7c,d) show a similar behavior: increasing the concentration of Zr leads to longer interatomic distances between the absorber and tetravalent cations, while the distortion parameters are unchanged. In particular, the values of σ^2^ for Gd-Ti are the highest, reaching 0.05 Å^2^ in GTZ_0_, showing once again the extreme disorder of the amorphous sample even at the local scale.

Only GTZ_100_ was analyzed at both Zr K- and Gd L_3_-edges. As already mentioned in the beginning of this section, using the same k and R intervals allows us to conduct a direct comparison between common parameters, specifically the interatomic distance and the distortion parameter for Gd-Zr in this sample. As can be seen in Figure 6c or Figure 7c and in Table S3, the values derived from the Zr K- and Gd L_3_- spectra, which are σ^2^ = 0.014 (3) and 0.015 (3) and R = 3.64 (3) Å and 3.61 (3) Å, respectively, are in good agreement. The complete EXAFS investigation of this sample enables direct comparison with the PDF results. As already noted, the Zr-O and Gd-O distances calculated from EXAFS analysis accurately match with the positions of the two *G*(*r*) peaks in the 2–2.5 Å range. Moving to cation–cation interatomic distances, according to EXAFS analysis, they span from 3.51 Å (Zr-Zr) to 3.69 Å (Gd-Gd). According to PDF analysis, the same cation–cation interatomic distances span in the 3.53–3.91 Å range, with an average value of ≈3.7 Å. The range of interatomic distances detected by PDF seems to be larger and broader than those detected by EXAFS. This apparent mismatch can be rationalized by considering that, on the one hand, the distribution of distances detected by EXAFS should be broader than 3.51–3.69 Å, given the high σ^2^ values calculated for these distances (≈0.015 Å^2^), and, on the other hand, the distances calculated by PDF derive from fitting data over a wide range of interatomic distances (1.9–8.0 Å) using the Weberite structure, and thus represent a middle-range order (within ≈1 nm) rather than a local one.

### 3.4. General Discussion

Based on the obtained experimental results and their comparison with structural data from well-grown crystalline phases (solid-state synthesis at 1500 °C) of the same solid solution in our previous work [11], we propose the following general discussion. The average structure of the latter samples is pyrochlore across the whole compositional range. While the pure Gd_2_Ti_2_O_7_ compound is almost defect-free, increasing Zr concentration promotes the growth of Anti-Frenkel oxygen defects and the partial swapping of Gd^3+^/Zr^4+^ ions. Conversely, the present sol–gel samples show complex behavior. HR-XRPD data revealed that samples with low Zr content (xZr ≤ 0.25) are amorphous. Increasing Zr concentration led to the emergence of a second crystalline phase, identified as defective fluorite (xZr = 0.375, 0.56). Samples with the highest Zr content (xZr ≥ 0.75) are fully crystalline and exhibit only the fluorite phase.

W-H (and PDF) analysis detected the nanosize nature of the sol–gel samples: *D*_V_ spans from ≈1.5 nm for the GTZ_37.5_ sample to ≈4 nm for the GTZ_100_ one.

When xZr is reduced, the cell parameter *a_F_* shrinks, consistent with the smaller ionic radius of Ti^4+^ with respect to Zr^4+^. However, the cell parameters of the nanocrystalline samples do not follow a linear trend. In particular, transitioning from fully crystalline samples to partially amorphous ones, the cell parameter tends to a constant value, suggesting that the biphasic samples are inhomogeneous both crystallographically and compositionally, with the amorphous phase being richer in Ti than the average composition, and vice versa for the nanocrystalline one. Accordingly, the amorphous background of both biphasic samples closely matches with the diffraction pattern of GTZ_25_, as displayed in Figure S3.

Under the hypothesis suggested by the literature that the sol–gel samples fired at relatively low *T* values (500 °C) should mimic the radiation damage of initially crystalline compounds, the present results confirm that Gd_2_Ti_2_O_7_ tends to become amorphous. The amorphous phase may include at least 12.5% of Zr^4+^ ions (with respect to the total number of cations). For larger xZr values, part of the zirconium ions segregates, thus forming a Zr-rich nanophase. The same phenomenon can be observed starting from the opposite side. The fluorite structure of the GTZ_100_ samples is consistent with the literature: this sample can dissipate kinetic energy by forming Anti-Frenkel defects and mixing the Zr/Gd positions, thanks to the low formation enthalpy of such defects, thereby facilitating the pyrochlore → fluorite phase transition. Again, sample homogeneity is not affected only for a relatively small Ti concentration (1-xZr ≤ 0.25).

A further step was to map the structure of the formed defective phases, either crystalline or amorphous, using local probes such as the PDF analysis of diffraction data and EXAFS investigation.

The experimental *G*(*r*) functions displayed two or three peaks in the 1.8–2.5 Å range, corresponding to the nearest-neighbor cation–oxygen distances. A first peak at *r* ≈ 1.9 Å appears only for the sample with the highest Ti content; a second peak, whose intensity increase with increasing xZr, is centered at *r* ≈ 2.1 Å, and a third peak at *r* ≈ 2.35 Å is almost unaffected by sample compositions. EXAFS analysis allows us to attribute these three peaks to Ti-O [24], Zr-O, and Gd-O distances, respectively. The small changes in these *r* values across the whole compositional range suggest that the local environments of cations are similar in both the crystalline and the amorphous phases.

Moving to the shortest cation–cation distances, according to the defect fluorite model, they should all drop at ≈3.7 Å. Conversely, all the investigated biphasic and fluorite (on average) samples display a *G*(*r*) peak centered at ≈3.62 Å with a tail on its right side. Both the position of the peak maximum and the presence of the tail are inconsistent with the fluorite structure. When lowering the Zr concentration, the peak at ≈3.62 Å, as well as all the cation–cation distances, broadens, thus enforcing the idea that disorder increases in Ti-rich samples.

EXAFS confirmed all these results. Focusing on the GTZ_100_ sample, investigated at both the Zr K- and Gd L_3_-edges, different cation–cation interatomic distances were found, spanning from 3.51 Å (Zr-Zr) to 3.69 Å (Gd-Gd). This finding is incompatible with the defect fluorite structure involving 12 identical cation–cation distances for each cation.

Furthermore, the analysis of data collected at the Gd L_3_-edge encompassing the whole compositional range revealed that the distortion parameter σ^2^ for both Gd-O and Gd-Gd distances decreases significantly from the fully amorphous (GTZ_0_) to the fully crystalline phase, which matches with the broadening trend of the *G*(*r*) peaks following the same compositional coordinate.

Thus, decreasing crystal dimension (and, based on the cited literature [24], the occurrence of radiation damage) introduces much more disorder in Ti-rich compounds. In fact, the Gd_2_Zr_2_O_7_ compound can accommodate complex defect clusters by increasing the coordination number of Zr^4+^ [11]. Conversely, the defect formation enthalpy in Gd_2_Ti_2_O_7_ is higher than the previous case, and titanium ions hardly change their ideal coordination number (=6). Without an easy way to dissipate the energy induced by irradiation, these events could affect the integrity of the crystalline structure.

A third step, limited to the fully crystalline GTZ_75_ and GTZ_100_ samples, consisted of modeling the short-range (1.9–8.0 Å) disorder. Attempts to fit the data using either a defect fluorite phase or a pyrochlore model (that corresponds to the long-range structure of microcrystalline samples [11]) were ineffective, as expected, because both models imply 12 identical nearest-neighbor cation–cation distances.

Conversely, the Weberite *Ccmm* model, which allows for cation positional degrees of freedom, provided an acceptable fit to the *G*(*r*) data collected from both samples, suggests that, taking the defect fluorite structure as a reference, the tendency to allocate vacancies in cation’s first coordination shell follows the order Ti^4+^ > Zr^4+^ > Gd^3+^.

Finally, for *r* ≥ 9 Å, the same *G*(*r*) functions could be fitted using the defect fluorite model, setting an upper limit to the structural coherence length of the defect clusters and reconciling the short- and long-range structural findings.

## 4. Conclusions

In this work we investigated the structural evolution across different compositions of Gd_2_(Ti_1−x_Zr_x_)_2_O_7_ solid solutions (xZr = 0.00, 0.15, 0.25, 0.375, 0.56, 0.75, 0.85, 1.00) synthesized via the sol–gel method and thermally treated at 500 °C using HR-XRPD to assess long-range structure, while the local structure was assessed by and PDF analysis and EXAFS spectroscopy.

According to HR-XRPD data the samples with xZr ≤ 0.25 are amorphous; at intermediate Zr concentrations (xZr = 0.375, 0.56) the amorphous phase is flanked by a defective fluorite nanocrystalline phase while for xZr ≥ 0.75 the samples were fully crystalline and exhibited only the fluorite phase, the crystallographic coherence length being of the order of several nanometers

The experimental PDF of the fully crystalline samples (xZr ≥ 0.75) were suitably fitted by the defect fluorite model only for *r* > 9 Å, while misfits appear for smaller *r* values. In particular, the experimental *G*(*r*) peaks corresponding to cation–cation interatomic distances display asymmetries and high-r tails odd with the defect fluorite model.

The PDF/*G*(*r*) data in the 1.8 ≤ *r* ≤ 8.0 Å were modeled using Weberite structure (S.G. *CmCm*) which allows the relaxation of the cation coordinates and revealed that the tendency to allocate vacancies in cation first coordination shell is Ti^4+^ > Zr^4+^ > Gd^3+^.

EXAFS analysis at the Zr-K and Gd-L_3_ edges, confirms the splitting of the cation–cation distances associated with different metal species and provided a local structural description of the amorphous phases, identifying a consistent Gd-O distance across all compositions.

The findings of this study facilitate the structural characterization of matrices compromised by decay-induced damage, thereby establishing a rigorous framework for discerning optimal compositions for the immobilization of radioactive waste. Comparative assessment of the structures with fully crystalline samples from previous own work enables elucidation of the damage progression mechanisms, allowing identification of compositions exhibiting enhanced resistance to radiation-induced degradation, and thus representing the most viable candidates for deployment as durable waste form matrices.

## Figures and Tables

**Table 1:** Nominal and experimental Zr and Ti percentages obtained by EDX measurements. All data report the deviation calculated by averaging three area scans.

Sample	Nominal Zr %	Exp. Zr %	Nominal Ti %	Exp. Ti %
GTZ_15_	15	15.6 ± 1.3	85	87.8 ± 1.2
GTZ_25_	25	24.0 ± 0.4	75	81.1 ± 2.9
GTZ_37.5_	37.5	38.9 ± 1.6	62.5	59.8±
GTZ_56_	56	55.8 ± 1.6	44	44.1 ± 1.6
GTZ_75_	75	76.4 ± 2.4	25	23.5 ± 2.5
GTZ_85_	85	86.6 ± 0.7	15	13.4 ± 0.8

**Table 2:** The Rietveld refinements and W-H results obtained by employing space group $Fm\bar{3}m$, Gd/Zr/Ti 4a (0, 0, 0), and O 8a (¼, ¼, ¼).

Sample	GTZ_100_	GTZ_85_	GTZ_75_	GTZ_56_	GTZ_37.5_
xZr	1	0.85	0.75	0.56	0.35
*a_F_* (Å)	5.2590 (4)	5.2547 (4)	5.2473 (5)	5.2344 (4)	5.2326 (8)
U_ave_/Å^2^	0.0139 (3)	0.0129 (4)	0.0155 (4)	0.0099 (4)	0.0128 (8)
Rp	0.0342	0.0362	0.0346	0.0219	0.0553
R(F^2^)	0.0410	0.0339	0.0274	0.0562	0.0540
*D*_V_/Å	45 (3)	40 (4)	36 (5)	23 (3)	
ε	0.015 (1)	0.014 (2)	0.014 (3)	0.004 (3)	

## Data Availability

Data obtained during beamtime CH6375 are available at doi.esrf.fr/10.15151/ESRF-ES-1031243084, while data obtained during beamtime CH6416 are available at doi.org/10.15151/ESRF-ES-1031240739. Other data are available upon request to the authors.

## Supplementary Materials

The following supporting information can be downloaded at https://www.mdpi.com/article/10.3390/nano15141134/s1. Figure S1: SEM micrographs of the studied samples. Figure S2: EDX spectra of the studied samples. Figure S3: HR-XRPD pattern of GTZ_25_ and GTZ_56_. Table S1: PDF refinement results using the fluorite model in the 2–50 Å range. Figure S4: PDF fit in 2–50 Å range using the fluorite model. Figure S5: PDF fit of sample GTZ37.5. Figure S6: PDF fit of sample GTZ75 using the Weberite model. Table S2: Cation–cation bond length obtained from the Rietveld refinements of the Weberite model. Figure S7: XAS spectra and respective Fourier transform used for EXAFS fitting. Table S3: Results obtained from EXAFS data fitting.

## Abbreviations

The following abbreviations are used in this manuscript in order of usage:

HR-XRPD	High-Resolution X-ray Powder Diffraction
PDF	Pair Distribution Function
DFT	Density Functional Theory
EXAFS	Extended X-ray Absorption Fine Structure
XANES	X-ray Near Edge Absorption Spectroscopy
XAS	X-ray Absorption Spectroscopy
ESRF	European Synchrotron Radiation Facility
SEM	Scanning Electron Microscopy
EDX	Energy Dispersive X-ray Spectroscopy
W.H.	Williamson–Hall
