# Supplementary material for: Synchrotron-Based Structural Analysis of Nanosized Gd2(Ti1−xZrx)2O7 for Radioactive Waste Management
Source: Nanomaterials (Basel). 2025 Jul 21;15(14):1134. doi: 10.3390/nano15141134 (PMC12298962; doi:10.3390/nano15141134)
Supplement: Supplementary file 1 [file nanomaterials-15-01134-s001.zip › nanomaterials-3752607-supplementary.pdf]

Supporting Information

Synchrotron-Based Structural Analysis of Nanosized $Gd_2(Ti_{1-x}Zr_x)_2O_7$ for Radioactive Waste Disposal

Marco Pinna [1,*,†], Andrea Trapletti [1,†], Claudio Minelli [1], Armando di Biase [1], Federico Bianconi [1], Michele Clemente [1], Alessandro Minguzzi [1,2], Carlo Castellano [1,*] and Marco Scavini [1,*]

1 Dipartimento di Chimica, Università degli studi di Milano, Via Golgi 19, 20133 Milan, Italy; andrea.trapletti@unimi.it (A.T.); armando.dibiase.adb@gmail.com (A.d.B.); federico.bianconi@studenti.unimi.it (F.B.); michele.clemente1@studenti.unimi.it (M.C.); alessandro.minguzzi@unimi.it (A.M.)

2 Dipartimento di Energia, Politecnico di Milano, Via Lambruschini 4a, 20156 Milan, Italy

* Correspondence: marco.pinna@unimi.it (M.P.); carlo.castellano@unimi.it (C.C.); marco.scavini@unimi.it (M.S.)

† These authors contributed equally to this work.

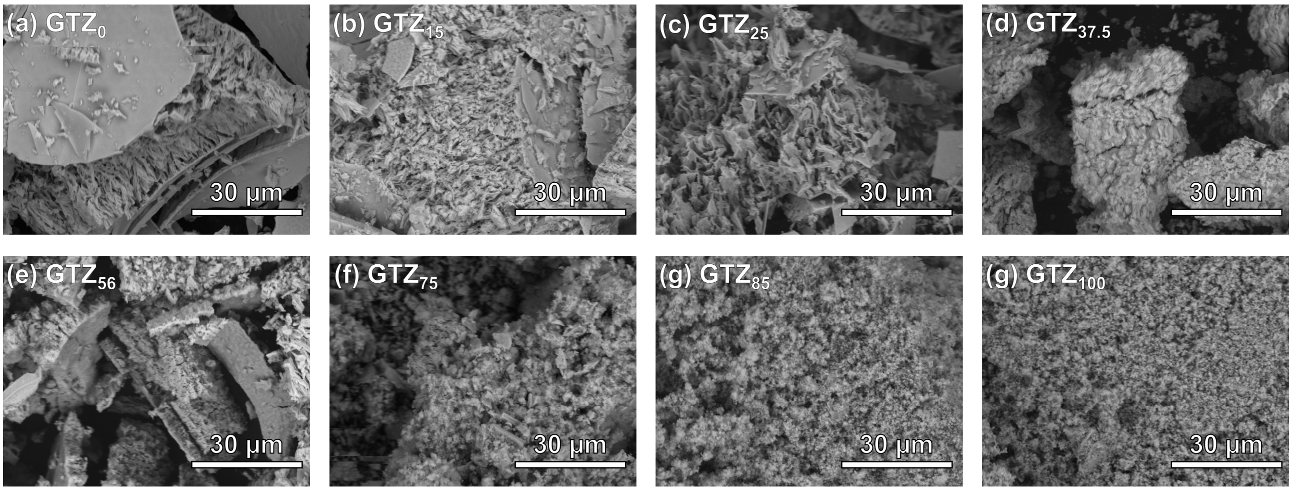

Figure S1: SEM micrographies for (a) GTZ$_0$, (b) GTZ$_{15}$, (c) GTZ$_{25}$, (d) GTZ$_{37.5}$, (e) GTZ$_{56}$, (f) GTZ$_{75}$, (g) GTZ$_{85}$, and (h) GTZ$_{100}$.

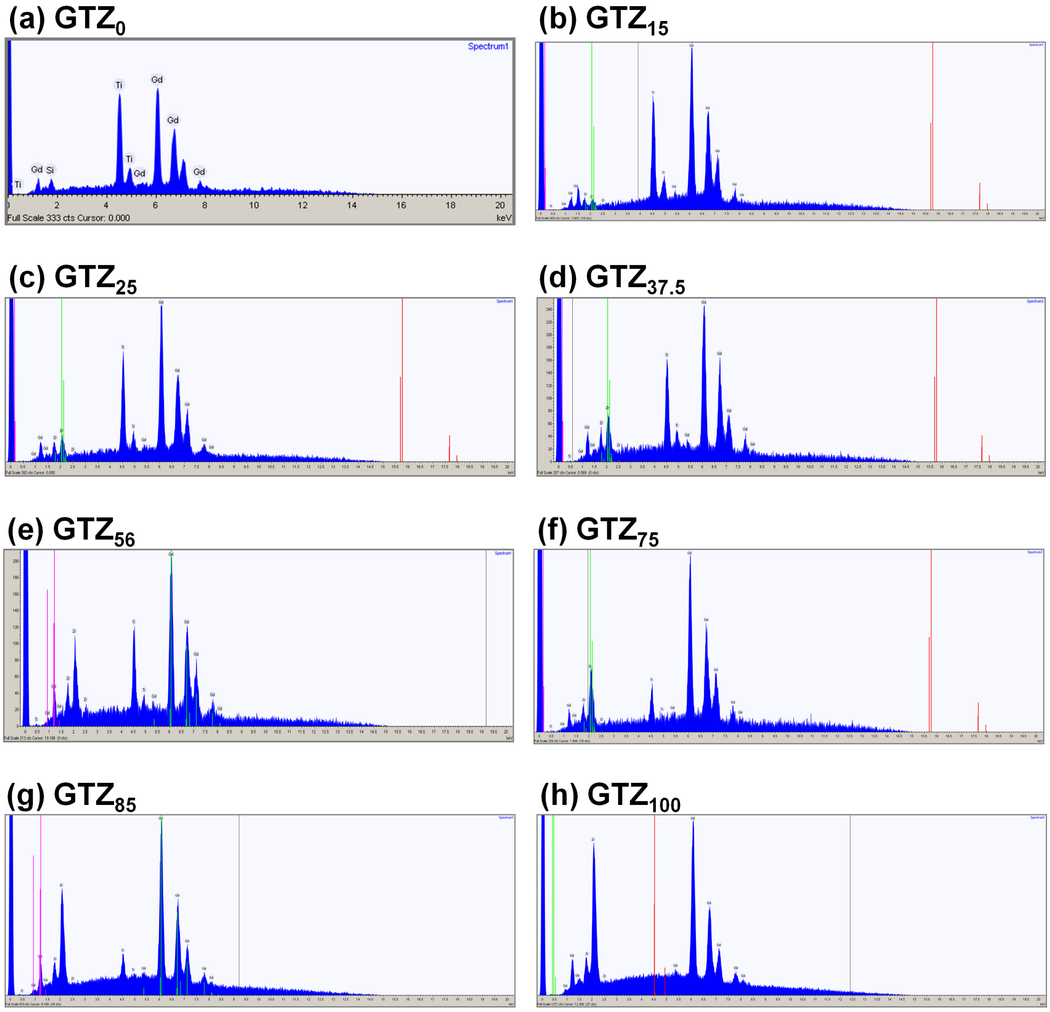

Figure S2: EDX spectra for (a) GTZ$_0$, (b) GTZ$_{15}$, (c) GTZ$_{25}$, (d) GTZ$_{37.5}$, (e) GTZ$_{56}$, (f) GTZ$_{75}$, (g) GTZ$_{85}$, and (h) GTZ$_{100}$.

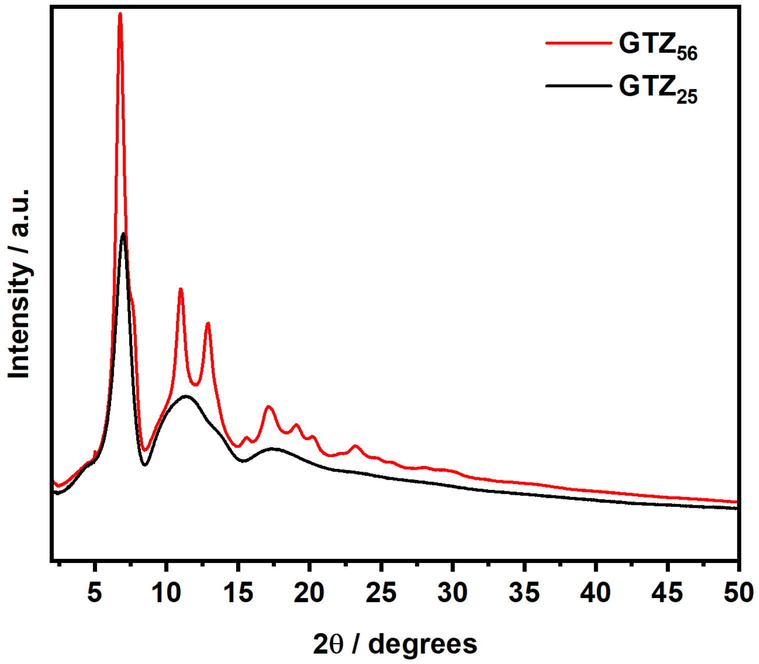

Figure S3: HR-XRPD patterns for GTZ_{25} (black line), GTZ_{56} (red line) used for the subtraction procedure.

Table S1: PDF refinements results using the Fluorite model (space group $Fm\bar{3}m$) in the 2-50 Å range. Atom positions: Zr/Gd 4a (0, 0, 0), O 8c (¼, ¼, ¼).

	GTZ_{100}	GTZ_{75}	GTZ_{56}	$GTZ_{37.5}$
**Lattice parameter (*a*) / Å**	5.255(2)	5.244(2)	5.235(5)	5.24(1)
U_M	0.032(1)	0.0316(1)	0.044(3)	0.0609(5)
U_O	0.147(1)	0.171(2)	0.22(4)	0.21(4)
D_V/Å	39.2(8)	35.1(9)	26(1)	16.1(7)
R_w	0.236	0.232	0.326	0.381

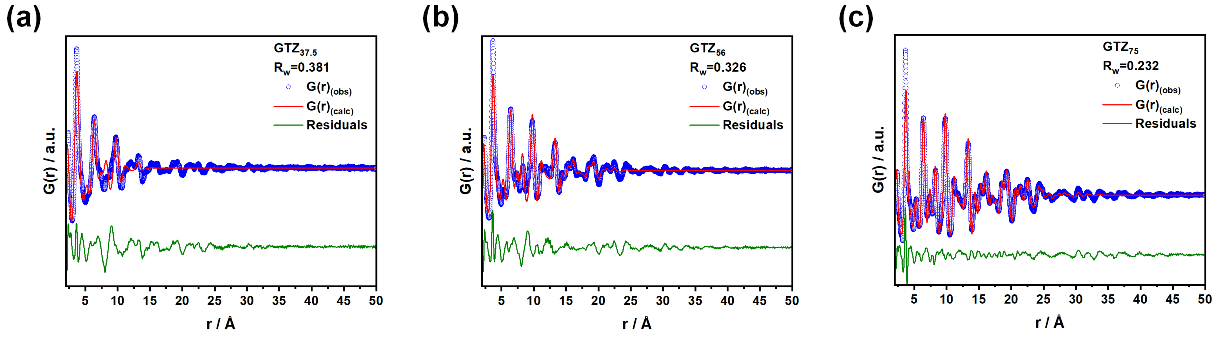

Figure S4: PDF fitting in the 2-50 Å region for (a) $GTZ_{37.5}$, (b) GTZ_{56}, and (c) GTZ_{75}.

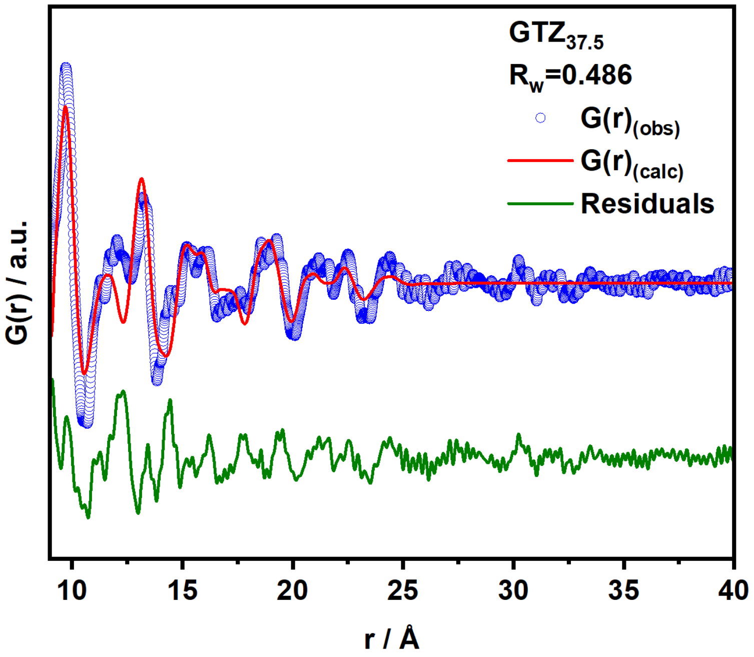

Figure S5: PDF fit for sample $GTZ_{37.5}$.

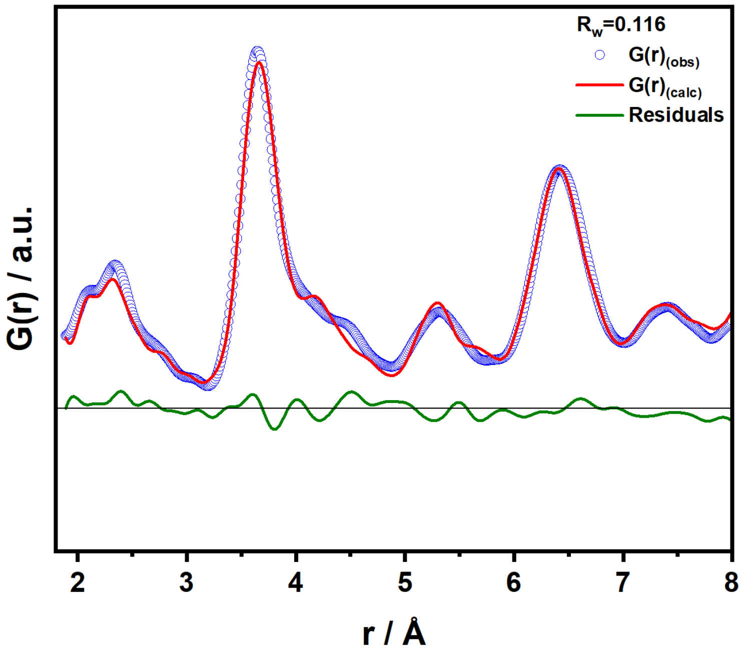

Figure S6:PDF fit for the sample GTZ75 using the Weberite model.

Table S2: Cation-cation bond length obtained from Rietveld refinements using the Weberite model. Wyckoff positions are Zr/Ti 4a (0, 0, 0), Zr/Gd 8g (≈0.236, 0.236, ¼), Gd 4b (0, ½, 0). The values are reported in function of the Wyckoff position since the 8g Wycoff position 4a is fully occupied by Zr in $GTZ_{10}0$ and shared between Ti and Zr (50% each) in GTZ_{75}.

**GTZ_{100}**			
**Wyckoff position**		**Molteplicity**	**Bond length / Å**
4b	4b	2	3.5821
4b	8g	4	3.6521
4b	8g	4	3.7290
4b	4a	2	3.9180
8g	4b	2	3.6521
8g	4b	2	3.7290
8g	8g	1	3.6758
8g	8g	1	4.1596
8g	8g	2	3.6024
8g	4a	2	3.5317
8g	4a	2	3.8539
4a	8g	2	3.9177
4a	8g	4	3.5200
4a	8g	4	3.8539
4a	4a	2	3.5821
**GTZ75**			
**Wyckoff position**		**Molteplicity**	**Bond length / Å**
4b	4b	2	3.5965
4b	8g	4	3.6485
4b	8g	4	3.7290
4b	4a	2	3.9013
8g	4b	2	3.6485
8g	4b	2	3.7290
8g	8g	1	3.7046
8g	8g	1	4.0980
8g	8g	2	3.6117
4a	4b	2	3.9013
4a	8g	4	3.5417
4a	8g	4	3.8305
4a	4a	2	3.5965

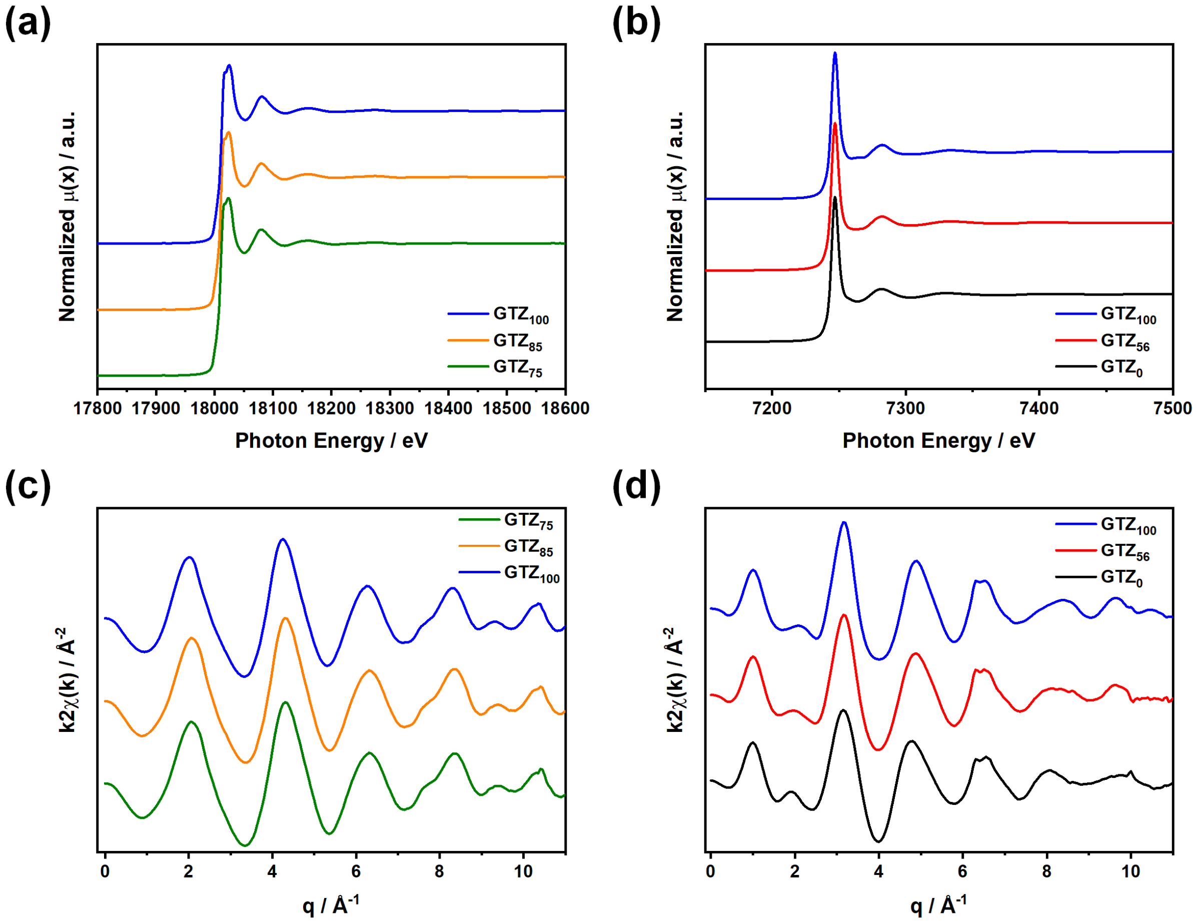

Figure S7: Absorption spectra and respective EXAFS signals at the (a,c) Zr K-edge and (b,d) Gd L_3-edge.

Table S3: Results obtained from EXAFS data fitting.

Zr K-edge				
		75	85	100
Zr-O	N	7	7	7
	S_0^2	1	1	1
	σ^2	0.0083 ± 0.0006	0.0081 ± 0.0007	0.0080 ± 0.0009
	R	2.15 ± 0.01	2.15 ± 0.01	2.14 ± 0.02
Zr-Zr	N	6	6	6
	S_0^2	0.75	0.85	1
	σ^2	0.012 ± 0.004	0.015 ± 0.007	0.014 ± 0.004
	R	3.60 ± 0.04	3.57 ± 0.04	3.5088 ± 0.03316
Zr-Gd	N	6	6	6
	S_0^2	1	1	1
	σ^2	0.019 ± 0.006	0.018 ± 0.006	0.014 ± 0.003
	R	3.61 ± 0.03	3.64 ± 0.04	3.64 ± 0.03
Zr-Ti	N	6	6	-
	S_0^2	0.25	0.15	-
	σ^2	0 ± 0	0 ± 0	-
	R	3.64 ± 0.03	3.63 ± 0.05	-
Gd L_3-edge				
		0	56	100
Gd-O	N	7	7	7
	S_0^2	1	1	1
	σ^2	0.0125 ± 0.0008	0.0116 ± 0.0008	0.011 ± 0.001
	R	2.35 ± 0.01	2.36 ± 0.01	2.36 ± 0.01
Gd-Gd	N	6	6	6
	S_0^2	1	1	1
	σ^2	0.03 ± 0.01	0.018 ± 0.007	0.015 ± 0.004
	R	3.5 ± 0.1	3.63 ± 0.06	3.69 ± 0.03
Gd-Zr	N	-	6	6
	S_0^2	-	0.56	1
	σ^2	-	0.014 ± 0.005	0.015 ± 0.003
	R	-	3.57 ± 0.03	3.61 ± 0.02
Gd-Ti	N	6	6	-
	S_0^2	1	0.44	-
	σ^2	0.05 ± 0.04	0.03 ± 0.04	-
	R	3.5 ± 0.1	3.6 ± 0.1	-
